# Loss of miR-26b-5p promotes gastric cancer progression via miR-26b-5p-PDE4B/CDK8-STAT3 feedback loop

**DOI:** 10.1186/s12967-023-03933-x

**Published:** 2023-02-03

**Authors:** Tingting Xu, Mengyan Xie, Xinming Jing, Huning Jiang, Xi Wu, Xinzhu Wang, Yongqian Shu

**Affiliations:** 1 Department of Oncology, The First Affiliated Hospital of Nanjing Medical University, Nanjing, China; 2 Department of Oncology, Gusu School, Suzhou Municipal Hospital, The Affiliated Suzhou Hospital of Nanjing Medical University, Suzhou, China; 3 Department of Oncology, Sir Run Run Hospital, Nanjing Medical University, Nanjing, China; 4 Jiangsu Key Lab of Cancer Biomarkers, Prevention and Treatment, Collaborative Innovation Center for Cancer Personalized Medicine, Nanjing Medical University, Nanjing, China

**Keywords:** miR-26b-5p, Inflammation, Gastric cancer, STAT3

## Abstract

**Background:**

Chronic inflammation is a well-known risk factor for the development of gastric cancer (GC). Nevertheless, the molecular mechanisms underlying inflammation-related GC progression are incompletely defined.

**Methods:**

Bioinformatic analysis was performed based on data from The Cancer Genome Atlas (TCGA) and Gene Expression Omnibus (GEO), and the expression of miR-26b-5p in GC cells and tissues was validated by quantitative real-time PCR (qRT-PCR). Cell proliferation was examined through Cell Counting Kit-8 (CCK8), 5-Ethynyl-2’-deoxyuridine (EdU), colony formation, flow cytometry, and tumor xenografts. Correlation between miR-26b-5p and Cyclin dependent kinase 8 (CDK8) or Phosphodiesterase 4B (PDE4B) was analyzed by dual-luciferase reporter assays, qRT-PCR, and Western blot. The effect of miR-26b-5p on the Signal transducer and activator of transcription 3 (STAT3) pathway was investigated using Western blot, immunofluorescence (IF), and immunohistochemistry (IHC). The impact of STAT3 on miR-26b-5p was determined by dual-luciferase reporter assays and qRT-PCR.

**Results:**

The expression of miR-26b-5p was significantly downregulated in *Helicobacter Pylori (H. pylori*)-infected GC cells. The decreased expression of miR-26b-5p was also detected in GC cells and tissues compared to normal gastric epithelium cells (GES1) and normal adjacent gastric tissues. The low expression of miR-26b-5p promoted GC proliferation in vitro and in vivo and was related to the poor outcome of GC patients. In terms of mechanism, miR-26b-5p directly targeted PDE4B and CDK8, resulting in decreased phosphorylation and nuclear translocation of STAT3, which was associated with the regulation of GC proliferation by miR-26b-5p. Notably, miR-26b-5p was transcriptionally suppressed by STAT3, thus forming the miR-26b-5p-PDE4B/CDK8-STAT3 positive feedback loop.

**Conclusion:**

The newly identified miR-26b-5p-PDE4B/CDK8-STAT3 feedback loop plays an important role in inflammation-related GC progression and may serve as a promising therapeutic target for GC.

**Supplementary Information:**

The online version contains supplementary material available at 10.1186/s12967-023-03933-x.

## Background

Gastric cancer (GC) is ranked the fifth leading cause of cancer-related mortality worldwide with a high rate of recurrence and unfavorable prognosis in advanced cases, remaining to be a serious public health burden to date [1]. Therefore, it is urgent to clarify the mechanisms underlying the progression of GC to identify promising targets for therapy.

Although the pathogenesis of GC has not been fully elucidated, a casual correlation has been established between GC and chronic inflammation, as is characterized by chronic gastritis induced by persistent infection with *Helicobacter Pylori (H. pylori)*, a type of spiral-shaped Gram-negative bacteria that colonizes gastric mucosa [2, 3]. Cytokines, such as Interleukin (IL)-6 and IL-8, are known to be up-regulated by *H. pylori* colonization or by the direct action of its virulence factors [4] and play a crucial role in contributing to chronic inflammation and sustaining aberrant growth and survival of cancer cells [5, 6]. IL-6 exerts its function by binding to its specific receptor α-subunits, IL-6Rα, which heterodimerizes with the common signal-transducing receptor β-subunit gp130, thus, resulting in the stimulation of several inflammatory signaling pathways including Signal transducer and activator of transcription 3 (STAT3). The STAT3, once activated by IL-6, is phosphorylated and translocates to the nucleus where it regulates the transcription of target genes related to the cell cycle or cell survival by binding to their regulatory elements [7, 8]. The constitutively activated STAT3 is involved in promoting a protumorigenic chronic inflammatory microenvironment and the progression of tumors of epithelial origin including GC [9, 10]. Nevertheless, the molecular bases for the persistent STAT3 activity in GC have not been fully understood.

MicroRNAs (miRNAs) are a class of evolutionarily conserved short non-coding RNAs, approximately 23 nucleotides in size that post-transcriptionally suppress the translation of or trigger the degradation of target genes through recognizing and binding to complementary target sites in the 3’-untranslated regions (3’UTR) [11, 12]. With the development of microarray techniques, the dysregulation of miRNA expression has been implicated in various tumor activities [13]. MiR-26b-5p is known as a key tumor suppressor in diverse types of cancers including hepatocellular carcinoma [14–16], breast cancer [17, 18], colorectal cancer [19], bladder cancer [20], and prostate cancer [21]. Moreover, increasing evidence suggests that miR-26b-5p regulates several inflammatory signal transduction pathways. For example, miR-26b-5p is reported as a potent inhibitor of the nuclear factor kappa-B (NF-κB) pathway by targeting TGF-beta activated kinase 1 (TAK1) and TGF-beta activated kinase 1 binding protein 3 (TAB3) in hepatocellular carcinoma [14]. Also, in hepatocellular carcinoma, lncRNA DLGAP1 antisense RNA 1 (DLGAP1-AS1) activates Janus kinase 2 (JAK2)/STAT3 signaling pathway by sponging miR-26a-5p and miR-26b-5p [15]. However, little is known about the specific effect of miR-26b-5p on the mechanism of inflammation-related GC progression.

Here, we presented evidence that tumor suppressor miR-26b-5p was significantly downregulated in GC cells and tissues, as well as under *H. pylori* infection. The low expression level of miR-26b-5p promotes GC proliferation in vitro and in vivo. We, therefore, hypothesized that miR-26b-5p was involved in the inflammation-related GC progression. To test the hypothesis, we explored the molecular mechanisms of miR-26b-5p-mediated GC proliferation by detecting the downstream effectors of miR-26b-5p. In this study, we demonstrated that the association of miR-26b-5p with Phosphodiesterase 4B (PDE4B) and Cyclin dependent kinase 8 (CDK8) and STAT3 might provide a possible explanation for the constitutive activation of the STAT3 in GC and provided new insight into the underlying mechanism of inflammation-related GC progression.

## Materials and methods

### Cell lines and tissue samples

Human gastric cancer cells, MGC803, HGC27, BGC823, and SGC7901, human embryonic kidney cells (HEK-293 T), and normal gastric epithelium cells (GES1) were purchased from the Chinese Academy of Sciences (Shanghai, China). The cells were cultured in RPMI 1640 medium (Gibco) supplemented with 10% fetal bovine serum (FBS) (ScienCell), 1% penicillin, and 1% streptomycin, and were incubated at 37 °C in a humidified atmosphere with 5% CO_2_. Human recombinant IL-6 (R&D Systems) was dissolved in sterile 1 × Phosphate Buffered Saline (PBS). A total of 10 pairs of GC tissues and adjacent normal tissues were obtained from patients with GC at the First Affiliated Hospital of Nanjing Medical University. This study was approved by the Ethics Committee of the First Affiliated Hospital of Nanjing Medical University. Informed consent was signed by all participants.

### *H. pylori* strains

In this study, *H. pylori* strain 26695 (ATCC 700392, CagA + /VacA −) was used for cell lines. Bacteria were inoculated on trypticase soy agar with 5% sheep blood agar plates (BD Biosciences) under microaerophilic conditions (5% O_2_, 10% CO_2_, and 85% N_2_) at 37 °C. Before *H. pylori* infection, bacteria were cultured in Brucella broth (BB, BD Biosciences) with 10% FBS (Atlanta Biologicals) under microaerophilic conditions at 37 °C for 2 days. The bacterium was harvested and co-cultured with GES1 or HGC27 cells at the indicated multiplicity of infection (MOI). The *H. pylori*-infected GES1 or HGC27 were incubated and collected for either 0, 3, 6, or 12 h.

### Transfection

MiRNA mimics and inhibitor were designed and synthesized by GenePharma. Small interfering RNAs (siRNAs) targeting CDK8 or PDE4B were designed and produced by RiboBio. Expression vectors encoding human PDE4B or CDK8 were synthesized and cloned into the vector pcDNA3.1 by GenePharma. MiR-26b-5p agomir, miR-26b-5p antagomir, and negative controls were produced by GENECHEM. The lentiviruses were added into GC cells, and stable cell lines were obtained by selection with puromycin. The miRNA mimics, inhibitor, siRNAs, and plasmid vectors were transfected into cells using Lipofectamine^™^ 3000 (Invitrogen) according to the manufacturer’s protocol. After 48 h of transfection, cells are harvested for subsequent RNA and protein extraction. The target sequences are listed in Additional file 7: Table S2.

### Dual-luciferase reporter assay

HEK-293 T cells (6 × 10^4^ cells per well) were seeded in 24-well plates, followed by co-transfection with luciferase reporter plasmids (pmirGLO) containing miR-26b-5p-CDK8/PDE4B binding sequences or mutant sequences and miR-26b-5p mimics or control mimics using Lipofectamine^™^ 3000 (Invitrogen, MA, USA). After 24 h, cell lysate was collected to measure firefly luciferase (FL) and Renilla luciferase (RL) activity using a dual-luciferase reporter assay system (Promega) according to the manufacturer’s instructions. The relative ratio of FL/RL was used to normalize the difference in transfection efficiency.

### RNA Extraction and quantitative real-time PCR (qRT-PCR)

Total cellular RNA was extracted by the TRIzol reagent (Invitrogen). Isolated RNA was reversely transcribed with HiScript QRT SuperMix (Vazyme). qRT-PCR assays were carried out utilizing SYBR Green PCR Master Mix (Vazyme) with an ABI Prism 7900 562 Sequence detection system (Applied Biosystems). Glyceraldehyde-3-phosphate dehydrogenase (GAPDH) and U6 were used as internal controls for mRNA and miRNA expression, respectively. The primers were synthesized by Ribobio and are listed as follows: miR-26b-5p: F: GCCACGTTCAAGTAATTCAGGA R: CGCAGGGTCCGAGGTATTC U6: F: CTCGCTTCGGCAGCACA R: AACGCTTCACGAATTTGCGT GAPDH F: GGGAGCCAAAAGGGTCAT R: GAGTCCTTCCACGATACCAA PDE4B F: AACGCTGGAGGAATTAGACTGG R: GCTCCCGGTTCAGCATTCT CDK8 F: ACCTGTTTGAATACGAGGGCT R: TGCCGACATAGAGATCCCAGT.

### Western blot

Total proteins in cultured cells were extracted by lysis buffer (Thermo Fisher) with protease and phosphatase inhibitors. An Enhanced BCA Protein Assay Kit (Beyotime) was used to quantify protein levels. Proteins were electrophoretically separated on 10% SDS-PAGE gels (Epizyme) and then transferred to the polyvinylidene fluoride membranes (Millipore), which were blocked in 5% bovine serum albumin (BSA) at room temperature (RT) for 1 h and then incubated with primary antibodies at 4 °C overnight. The primary antibodies used for Western blot assay are listed: CDK8 (1:1000, Cell Signaling Technology, Cat#: 4101), PDE4B (1:1000, Cell Signaling Technology, Cat#: 72096S), STAT3 (1:1000, Abcam, ab68153), pSTAT3 (Tyr 705) (1:1000, Abcam, ab267373), GAPDH (1:1000, Beyotime, AF0006), and anti-α-tubulin (1:1000, Beyotime, AF0001). Incubation of the goat anti-rabbit (1:1000, Beyotime, A0208) or the goat anti-mouse secondary antibodies (1:1000, Beyotime, A0216) are followed after being washed with Tris-Buffered Saline and Tween 20 buffer three times.

### Immunofluorescence (IF) and immunohistochemistry (IHC)

MGC803 cells were fixed with 4% paraformaldehyde at RT for 30 min after being treated with IL-6(50 ng/mL). Subsequently, cells were permeabilized with 0.5% Triton X-100 for 30 min and were then blocked with 5% BSA for 1 h at RT. Then, cells were incubated with primary antibodies against pSTAT3 (Tyr 705) (1:1000, Abcam, ab267373) at 4 °C for 1216 h. After being washed with PBST three times, cells were then incubated with FITC-coupled second antibodies (1:500, Beyotime, A0562) for 1 h, followed by staining nuclei with DAPI for 15 min. Stellaris STED confocal microscope was adopted to observe stained cells. IHC was performed as previously reported [22] with antibodies against pSTAT3 (Tyr 705) (1:1000, Abcam, ab267373). Leica pathological slice scanner was used to capture images.

### Cell viability, 5-Ethynyl-2’-deoxyuridine (EdU), colony formation, and cell cycle assay

Cell viability was measured by using Cell Counting Kit-8 (CCK8) (Beyotime Biotechnology). Transfected MGC803 (3 × 10^3^/well) or HGC27 (2 × 10^3^/well) were seeded in 96-well plates. CCK8 solution was added to each well and then incubated for 1 h at the appointed time (day0, day1, day2, day3, day4). Absorbance values of cells were measured at the wavelength of 450 nm. EdU assays were carried out with a Cell-Light EdU DNA Cell Proliferation Kit (RiboBio). For colony formation assay, transfected GC cells (500 cells/well in 6-well plates) were incubated in the 10% FBS-supplemented medium at 37 °C, 5% CO_2_ for 2 weeks. Cells were counted after being fixed with 4% formaldehyde for 30 min and stained with 0.1% crystal violet for 1 h. For cell cycle analysis, target cells were harvested and fixed in 70% ethanol. Cells were stained with propidium iodide (Sigma) and RNase A (Sangon) for 30 min at 37 °C and were analyzed with a BD FACS Calibur flow cytometer using the Modfit software (BD).

### Nude mouse xenograft models

4-week-old male BALB/c mice were purchased from the Model Animal Research Center of Nanjing University, China. HGC27 cells stably transfected with miR-26b-5p agomir, miR-26b-5p antagomir, and their negative controls were harvested. 7 × 10^6^ HGC27 cells (100 μL) were injected subcutaneously into the right flank of mice. The tumor volumes and body weights of mice were measured every 2 or 3 days. Four weeks after injection, mice were sacrificed for collecting tumors. Tumors were weighed and used for H&E staining and qRT-PCR analysis. In vivo experiments with BALB/c mice xenotransplant model were conducted following the approval of the Institutional Animal Care and Use Committee of Nanjing Medical University.

### Bioinformatics analysis

Limma package processed by R software (version 3.6.1) was used to analyze differentially expressed miRNAs in the GSE108306 dataset obtained from the Gene Expression Omnibus (GEO) database (http://www.ncbi.nlm.nih.gov/geo/). MiRNA-seq data and clinical data of 434 primary GC tissues and 41 normal tissues were retrieved from the The Cancer Genome Atlas (TCGA)- Stomach adenocarcinoma (STAD) dataset. The target genes of miRNA were predicted by TargetScan (http://www.targetscan.org), miRWalk (http://mirwalk.umm.uni-heidelberg.de/), and miRDB (http://mirdb.org/). The correlation between the miR-26b-5p expression and target genes expression was evaluated by linear regression analysis. UALCAN [23] was utilized to explore the expression of miR-26b-5p targets across GC and normal tissues. Gene Set Enrichment Analysis (GSEA) was performed using the TCGA-STAD dataset to explore downstream pathways related to PDE4B overexpression. The c2.cp.kegg.v7.1.symbols.gmt was used as the reference gene sets. When p-value and FDR q value were both  < 0.05, the gene set was regarded to be significantly enriched.

### Statistical analysis

The results were expressed as mean ± standard deviation (SD) of at least three independent experiments. Statistical analysis was assessed by SPSS software and GraphPad Prism software. The normality of data distribution was analyzed by Shapiro–Wilk test. Data obeying equal variance and normal distribution between two groups was analyzed by Student’s t-test. The statistical significance among multiple groups was analyzed by one-way analysis of variance (ANOVA) followed by Tukey's post-hoc test. P < 0.05 was considered to be statistically significant.

## Results

### Identification of miR-26b-5p as a specific regulator in *H. pylori*-related GC

**Fig. 1:**
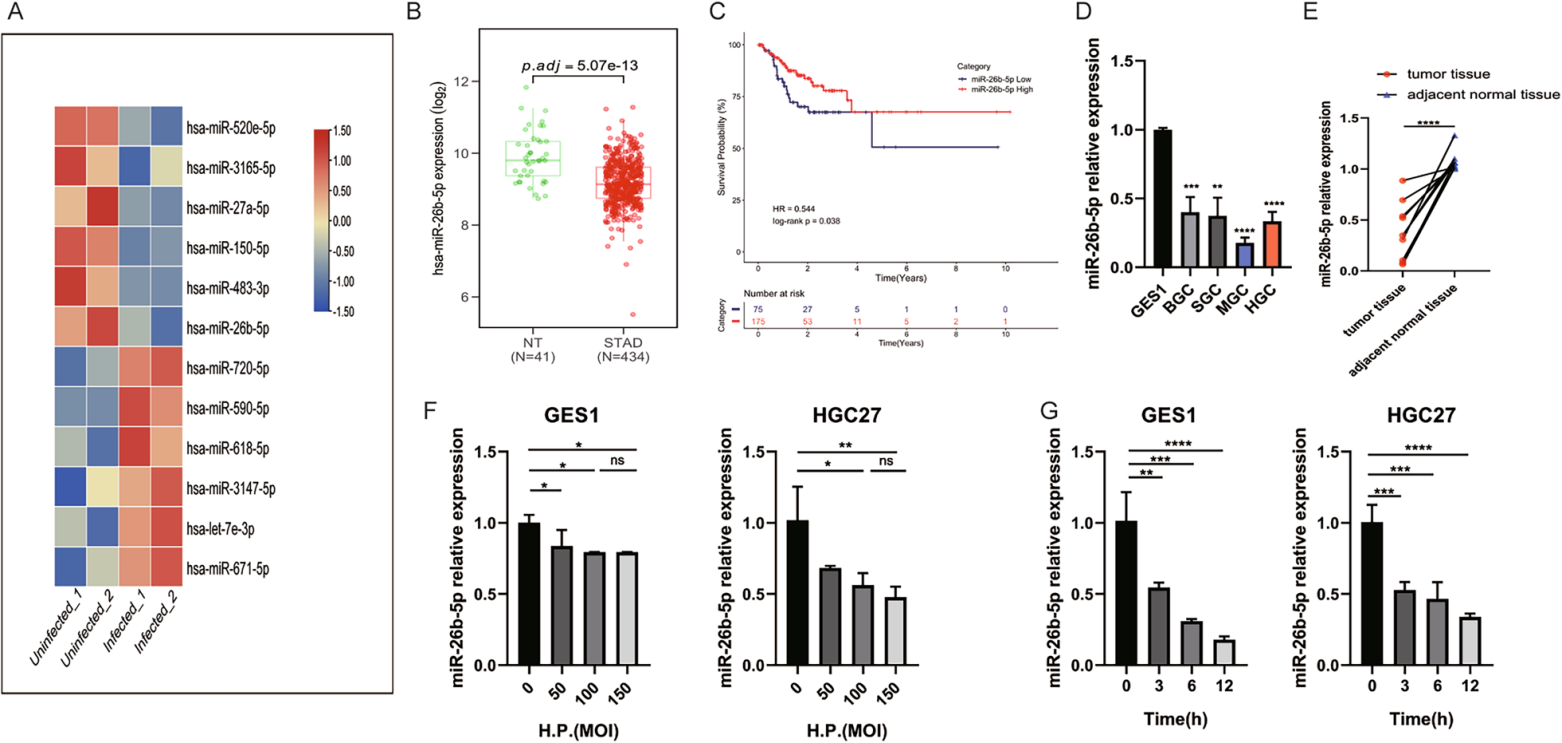
MiR-26b-5p is a candidate regulator of *H. pylori*-related GC. **A** Heatmap of dysregulated miRNAs in GSE108306. **B** The expression of miR-26b-5p in GC tissues (n = 434) and normal tissues (n = 41) was obtained from the TCGA database. **C** Kaplan–Meier analysis was conducted to assess the relationship between miR-26b-5p and the survival of GC patients. **D** MiR-26b-5p expression in GC cell lines and GES1 was detected by qRT-PCR. **E** MiR-26b-5p expression in 10 pairs of GC tissues and adjacent normal tissues using qRT-PCR. **F**–**G** qRT-PCR analysis of miR-26b-5p in GES1 and HGC27 cells infected with *H. pylori* with indicated MOIs for 6 h or with MOI 100:1 for indicated times. Quantitative data are shown as the mean ± SD of three independent experiments. *P < 0.05, **P < 0.01, ***P < 0.001, ****P < 0.0001

In order to identify miRNAs potentially dysregulated under *H. pylori* infection, we explored the differentially expressed miRNAs from GSE108306 between AGS cells infected and uninfected with *H. pylori* with the limma package of R language. In total, 12 miRNAs were selected by setting |log_2_foldchange(FC) |≥ 1.5 and P < 0.05 as the threshold (Fig. 1A). Moreover, based on TCGA data, among these miRNAs, we found that miR-26b-5p, which was downregulated under *H. pylori* infection, was poorly expressed in GC tissues (Fig. 1B). Also, ROC curve analysis showed that miR-26b-5p might serve as a diagnostic marker in GC, and the area under the curve (AUC) was 0.633 (p = 0.0049) in the TCGA-STAD dataset and 0.7155 (p = 0.0002) based on data from GSE30070 which includes 90 pre-treatment gastric cancer samples and 34 normal controls (Additional file 1: Figure S1). More importantly, Kaplan–Meier analysis indicated that low expression of miR-26b-5p was related to poor disease-free survival of GC patients (Fig. 1C). Then, we analyzed the expression of miR-26b-5p in GC cell lines and 10 pairs of GC tissues and adjacent normal tissues with qRT-PCR (Fig. 1D–E). The results indicated that miR-26b-5p was decreased in GC tissues and 4 GC cell lines (BGC823, SGC7901, HGC27, and MGC803), especially in HGC27 and MGC803 cell lines (HGC27: FC = 0.33, P < 0.0001; MGC803: FC = 0.18, P < 0.0001), hence, HGC27 and MGC803 cell lines were chosen for further experiments. Furthermore, to find out whether the expression of miR-26b-5p was regulated by *H. pylori* infection, miR-26b-5p expression was measured after co-culture of *H. pylori* 26695 with HGC27 and GES1 by qRT-PCR. The expression of miR-26b-5p decreased with *H. pylori* infection in a dose and time-dependent manner (Fig. 1F–G). Collectively, the aforementioned results indicate that miR-26b-5p is a potential prognostic marker for *H. pylori*-related GC.

### Suppression of miR-26b-5p promotes GC proliferation in vitro and in vivo

**Fig. 2:**
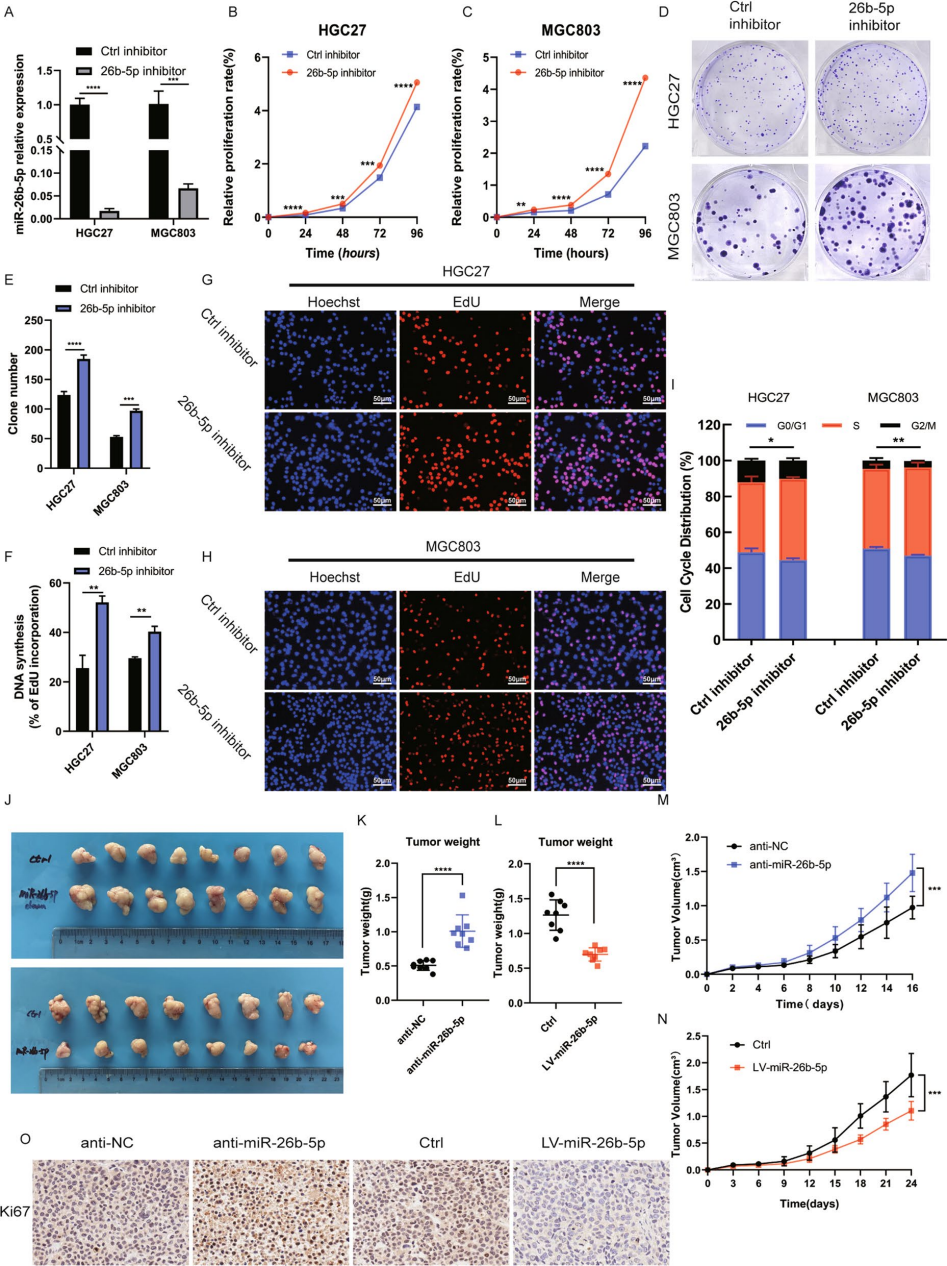
Suppression of miR-26b-5p promotes GC proliferation in vitro and in vivo*.* **A** qRT-PCR quantified the transfection efficiency of miR-26b-5p inhibitor in MGC803 and HGC27 cells. CCK8 **B**–**C**, colony formation **D**–**E**, and EdU **F**–**H** assays were conducted to assess the proliferation of MGC803 and HGC27 cells transfected with miR-26b-5p inhibitor or control. **I** Flow cytometry analysis of MGC803 and HGC27 cells transfected with miR-26b-5p inhibitor or control. **J** Pictures of xenograft tumors from nude mice 24 days after injection of miR-26b-5p agomir, miR-26b-5p antagomir, and their negative controls (LV miR-26b-5p, anti-miR-26b-5p, Ctrl, and anti-NC). **K**–**L** Tumor weight of xenografts was measured at the endpoint. **M**–**N** Tumor volume of xenografts was measured every 2 or 3 days and shown. **O** IHC of Ki67 in xenograft tumors. Quantitative data are shown as the mean ± SD of three independent experiments. *P < 0.05, **P < 0.01, ***P < 0.001, ****P < 0.0001

To explore the biological effect of miR-26b-5p on the proliferation capacity of GC, we first transfected miR-26b-5p inhibitor or mimics into HGC27 and MGC803 cells to knockdown (Fig. 2A) or overexpress (Additional file 2: Figure S2A) miR-26b-5p. CCK8, colony formation, and EdU assays revealed that miR-26b-5p knockdown notably promoted cell growth (Fig. 2B–H). Instead, cell proliferation in the miR-26b-5p overexpression group was significantly inhibited (Additional file 2: Figure S2B-H). Then we investigated the effects of miR-26b-5p on cell cycle using flow cytometry, the result showed that inhibition of miR-26b-5p decreased the population of G0/G1 phase cells, suggesting that G1/S cell-cycle transition is promoted by miR-26b-5p knockdown (Fig. 2I). In contrast, miR-26b-5p overexpression induced a significant G1-phase arrest and elevated the percentage of G0/G1 phase cells (Additional file 2: Figure S2I). What’s more, to confirm our results in a cell line with the higher basal expression of miR-26b-5p, we transfected the inhibitor of miR-26b-5p into BGC823 and then performed CCK8 and colony formation assays. It showed that suppression of miR-26b-5p promoted the proliferation of BGC823 (Additional file 3: Figure S3). Also, subcutaneous tumor models were established to examine the effect of miR-26b-5p in vivo*.* We found that tumors obtained from miR-26b-5p knockdown cells were significantly larger than control groups, both in terms of tumor volume and weight. Conversely, overexpression of miR-26b-5p was associated with a major reduction in tumor size and weight (Fig. 2J–N). Additionally, the IHC staining assay indicated that the expression levels of Ki67, a nuclear protein that is related to cellular proliferation, were decreased in the subcutaneous tumor tissues of mice in miR-26b-5p overexpression groups compared to control groups, while it showed the opposite result in miR-26b-5p knockdown groups (Fig. 2O). Collectively, these data indicate that miR-26b-5p is a potent inhibitor of GC cell proliferation in vitro and tumor growth in vivo.

### miR-26b-5p directly targets PDE4B and CDK8

**Fig. 3:**
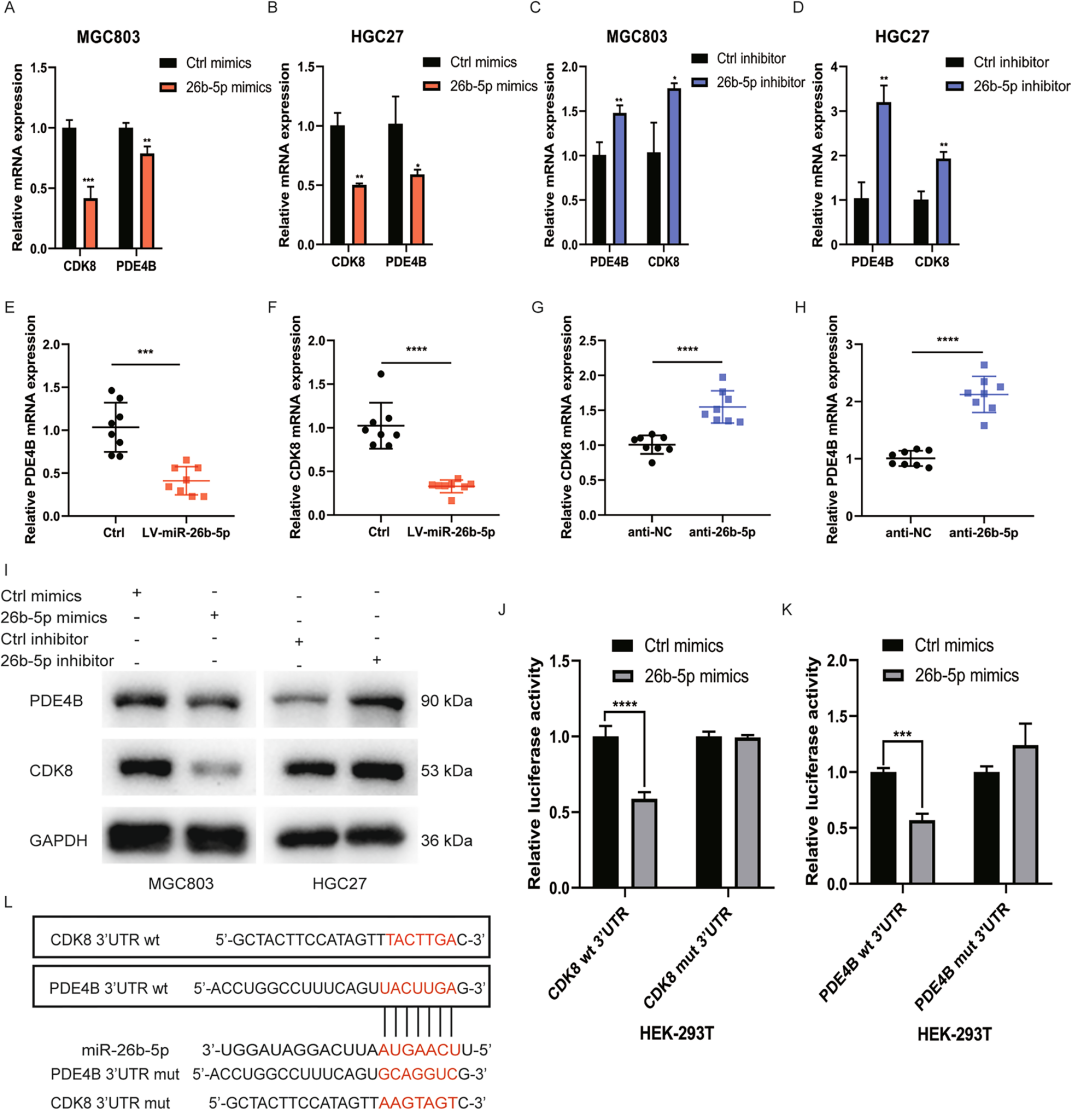
MiR-26b-5p directly targets PDE4B and CDK8. **A**–**B** qRT-PCR was conducted to analyze the expression of PDE4B and CDK8 in cells transfected with miR-26b-5p mimics or inhibitor **C**–**D** for 48 h. **E**–**F** PDE4B or CDK8 expression was detected in xenografts of nude mice injected with miR-26b-5p agomir or miR-26b-5p antagomir **G**–**H** using qRT-PCR. **I** Western blot analysis of PDE4B and CDK8 protein levels in cells transfected with miR-26b-5p mimics, inhibitor, or controls for 48 h. **J**–**K** Luciferase reporter was carried out in HEK-293 T cells co-transfected with dual-luciferase reporter genes containing the wild type (wt) or mutant (mut) CDK8/PDE4B 3’UTR and miR-26b-5p mimics or control mimics. **L** Schematic representation of putative wild or mutant miR-26b-5p binding sites in PDE4B/CDK8 3’ UTR. Quantitative data are shown as the mean ± SD of three independent experiments. *P < 0.05, **P < 0.01, ***P < 0.001, ****P < 0.0001

To explore the downstream mechanism by which miR-26b-5p inhibits GC progression, we utilized a three-step approach to identify potential targets of miR-26b-5p (Additional file 4: Figure S4A). Firstly, we searched for the possible targets of miR-26b-5p through three online predict databases TargetScan, miRDB, and miRWalk (Additional file 4: Figure S4B). Secondly, we analyzed the correlation between target genes and miR-26b-5p based on the TCGA database of GC by Spearman correlation analysis (P < 0.05, |cor|≥ 0.15). Thirdly, we examined the expression of these genes between tumor samples and normal samples in the UALCAN dataset (P < 0.05). Eventually, 16 protein-coding genes were chosen as candidates (Additional file 4: Figure S4C-D). After that, we transfected the mimics and inhibitor of miR-26b-5p into HGC27 and MGC803 cells and then detected the expression of these genes by qRT-PCR. The result showed that the expression of CDK8 and PDE4B was significantly decreased after transfection of miR-26b-5p mimics while inhibiting miR-26b-5p upregulated the expression of PDE4B and CDK8 (Fig. 3A–D). Western blot analysis confirmed that miR-26b-5p negatively regulated the expression of PDE4B and CDK8 proteins (Fig. 3I). We also investigated the expression of PDE4B and CDK8 in tumor tissues of nude mice described above. The expression of PDE4B and CDK8 mRNA was lower in the miR-26b-5p overexpression group compared to the control group, while knockdown of miR-26b-5p upregulated PDE4B and CDK8 mRNA expression, indicating that miR-26b-5p regulated PDE4B and CDK8 in vivo (Fig. 3E–H). Additionally, we constructed luciferase reporter plasmids containing 3’UTR of PDE4B or CDK8 with miR-26b-5p binding sites mutant or not, luciferase activity assays of 3′UTR-WT were remarkably reduced when miR-26b-5p was overexpressed in HEK-293 T cells, while no significant effect on the luciferase activity of 3’UTR-MUT was observed (Fig. 3J–L). Taken together, the results reveal that miR-26b-5p directly targets PDE4B and CDK8.

### miR-26b-5p modulates cells growth by targeting PDE4B and CDK8

**Fig. 4:**
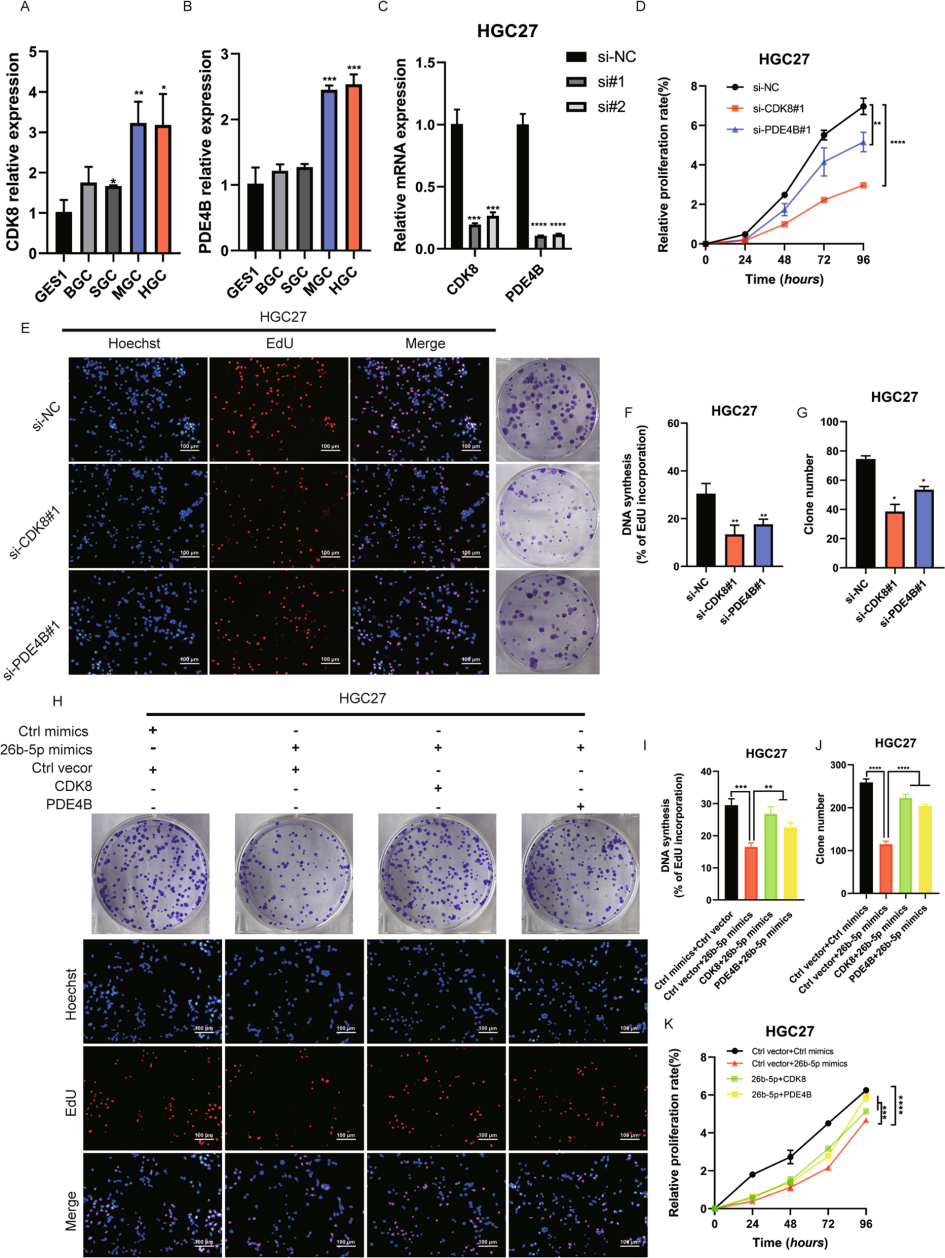
miR-26b-5p modulates cell growth by targeting PDE4B and CDK8. **A**–**B** Analyzing the expression of PDE4B and CDK8 in GC cell lines and GES1 cells by qRT-PCR. **C** Knockdown efficiency of PDE4B and CDK8 siRNAs. CCK8 **D**, colony formation, and EdU assays **E**–**G** were conducted to detect the effects of PDE4B and CDK8 silencing on HGC27 cell proliferation. HGC27 cells were transfected with miR-26b-5p mimics or miR-26b-5p mimics in combination with PDE4B or CDK8 overexpression vectors. Colony formation, EdU **H**–**J**, and CCK8 **K** assays were performed with the above cells to investigate whether PDE4B and CDK8 interfere with the function of miR-26b-5p. Quantitative data are shown as the mean ± SD of three independent experiments. *P < 0.05, **P < 0.01, ***P < 0.001, ****P < 0.0001

To further investigate the role of PDE4B and CDK8 in GC, we first examined the expression of PDE4B and CDK8 in GC cell lines using qRT-PCR. We found that PDE4B and CDK8 were highly expressed in GC cell lines compared to GES1, especially in HGC27 and MGC803, which was opposite to the expression of miR-26b-5p in GC cell lines suggesting that miR-26b-5p negatively regulates PDE4B and CDK8 (Fig. 4A–B). PDE4B or CDK8 siRNAs were transfected into MGC803 and HGC27 cells respectively to explore the biological functions of PDE4B and CDK8. Two independent siRNAs against PDE4B or CDK8 were utilized and we selected si-PDE4B#1 and si-CDK8#1 with the strongest knockdown efficiency for subsequent experiments (Fig. 4C). Functional experiments showed that silencing PDE4B or CDK8 inhibited cell proliferation which indicated a protumorigenic role for PDE4B and CDK8 (Fig. 4D–G, Additional file 5: Figure S5A-D). Furthermore, CCK8, colony formation, and EdU assays showed that miR-26b-5p-mediated suppression of cell proliferation was rescued by PDE4B or CDK8 overexpression in HGC27 cells (Fig. 4H–K). Similarly, PDE4B or CDK8 silencing impaired miR-26b-5p inhibition-induced MGC803 cells growth (Additional file 5: Figure S5E-H). Thus, our results demonstrate that miR-26b-5p attenuates the proliferation of GC by inhibiting PDE4B and CDK8.

### miR-26b-5p regulates the IL-6-induced STAT3 signaling pathway by targeting PDE4B and CDK8

**Fig. 5:**
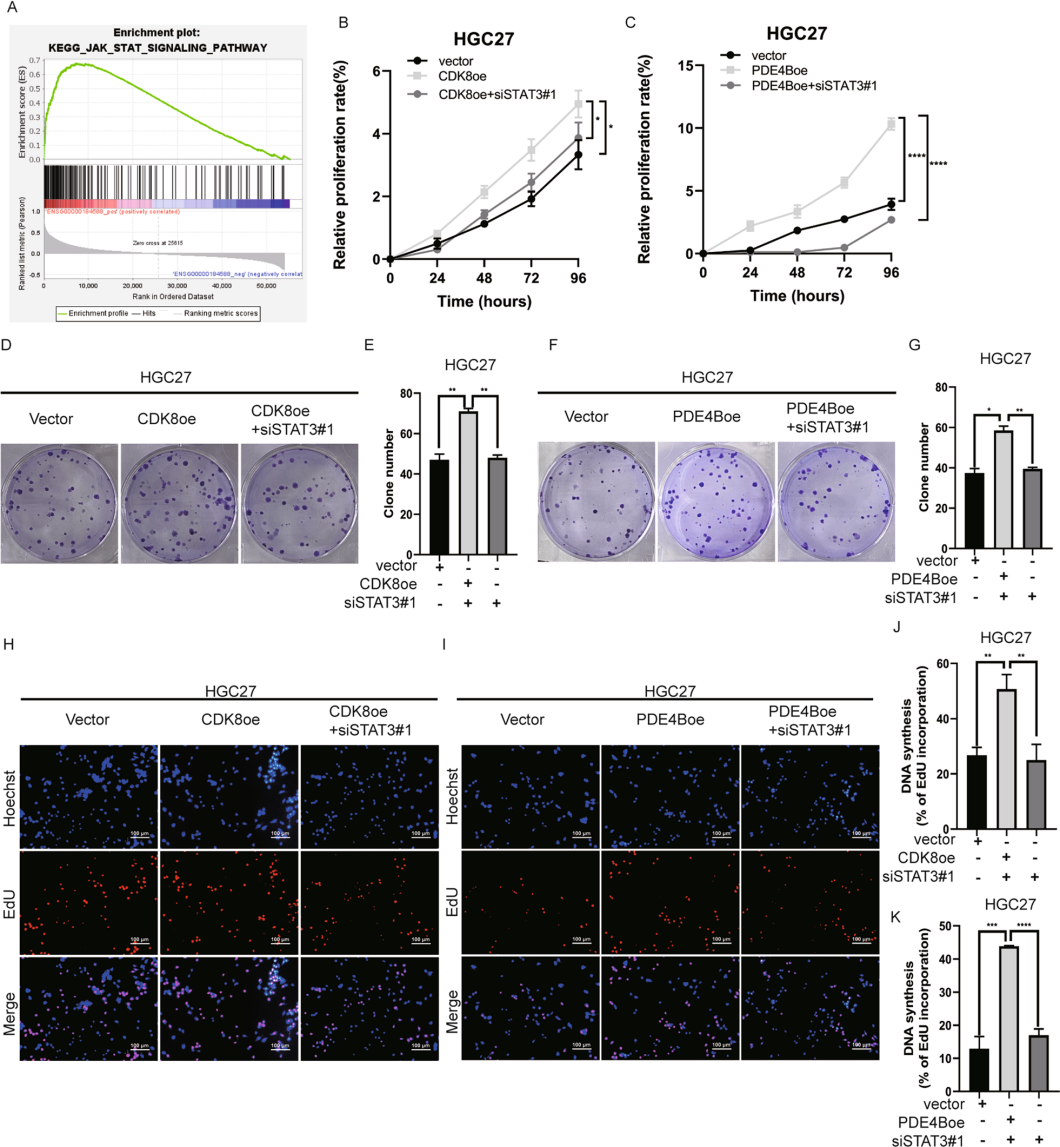
PDE4B and CDK8 regulate GC proliferation through STAT3. **A** GSEA using TCGA-STAD dataset. PDE4B overexpression was significantly associated with JAK/STAT signaling pathway. CCK8 (**B**), colony formation (**D**–**E**), and EdU (**H**, **J**) assays were conducted to assess the proliferation of HGC27 cells transfected with CDK8 overexpression plasmid or CDK8 overexpression plasmid plus STAT3 siRNAs. CCK8 (**C**), colony formation (**F**–**G**), and EdU (**I**, **K**) assays were performed to examine the proliferation of HGC27 cells transfected with PDE4B overexpression plasmid or PDE4B overexpression plasmid plus STAT3 siRNAs. Quantitative data are shown as the mean ± SD of three independent experiments. *P < 0.05, **P < 0.01, ***P < 0.001, ****P < 0.0001

It has been reported that PDE4B overexpression is associated with promoting the pathogenesis of hematologic malignancies and some solid tumors, such as colon cancer and bladder cancer [24, 25]. However, few studies have explored the exact role of PDE4B in GC. To gain more insight into the biological functions of PDE4B in GC, GSEA analysis of PDE4B was performed based on the TCGA-STAD dataset. It was found that the “JAK/STAT signaling” pathway was significantly enriched, suggesting that PDE4B overexpression was positively related to JAK/STAT signaling (Fig. 5A). Also, studies have shown that CDK8 was involved in phosphorylating STAT3 [26–29]. STAT3 siRNA and PDE4B or CDK8 overexpression plasmids were co-transfected into GC cells to investigate whether STAT3 is involved in the regulatory effect of PDE4B or CDK8 on GC cell proliferation. As expected, functional experiments showed that siSTAT3 reversed the proliferation-promoting effect of PDE4B or CDK8 overexpression in HGC27 (Fig. 5B–K) and MGC803 (Additional file 6: Figure S6A-J) cells, suggesting that PDE4B and CDK8 at least partially regulated cell proliferation through STAT3.

**Fig. 6:**
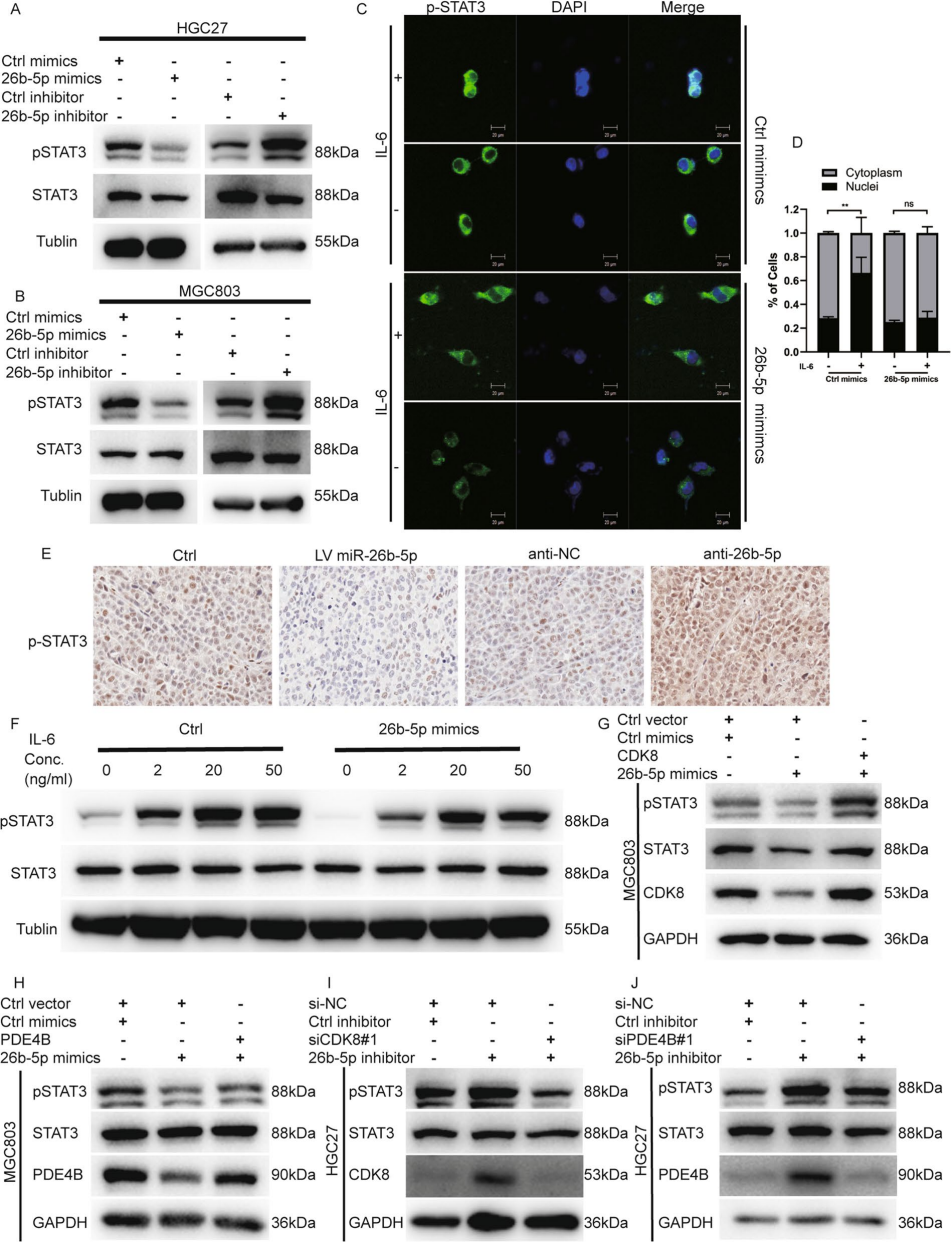
MiR-26b-5p regulates STAT3 signaling by targeting PDE4B and CDK8. **A**–**B** Total STAT3 expression and phosphorylation were detected by Western blot in MGC803 and HGC27 cells transfected with miR-26b-5p mimics, inhibitor, or controls. **C**–**D** MGC803 cells transfected with miR-26b-5p mimics or control mimics were treated with IL-6 (50 ng/mL) for 30 min before immunofluorescent staining for phosphorylated STAT3 (green). The nuclei were stained with DAPI (blue). **E** IHC of phosphorylated STAT3 in xenograft tumors of nude mice infected with miR-26 agomir, miR-26b-5p antagomir, or negative control cells (LV miR-26b-5p, anti-miR-26b-5p, Ctrl, and anti-NC). **F** MGC803 cells post-transfection with miR-26b-5p mimics or control mimics for 48 h were treated with different concentrations of IL-6 for 30 min. Total STAT3 expression and phosphorylation were examined by immunoblot. **G** MGC803 cells were transfected with miR-26b-5p mimics and CDK8 overexpression plasmids or empty vectors. **I** HGC27 cells were transfected with miR-26b-5p inhibitor and CDK8 siRNAs or siNC. CDK8, total STAT3, and STAT3 phosphorylation expression were detected by Western blot. **H** MGC803 cells were transfected with miR-26b-5p mimics and PDE4B overexpression plasmids or empty vectors. **J** HGC27 cells were transfected with miR-26b-5p inhibitor and PDE4B siRNAs or siNC. PDE4B, total STAT3, and STAT3 phosphorylation expression were examined using Western blot. Quantitative data are shown as the mean ± SD of three independent experiments. *P < 0.05, **P < 0.01, ***P < 0.001, ****P < 0.0001

**Fig. 7:**
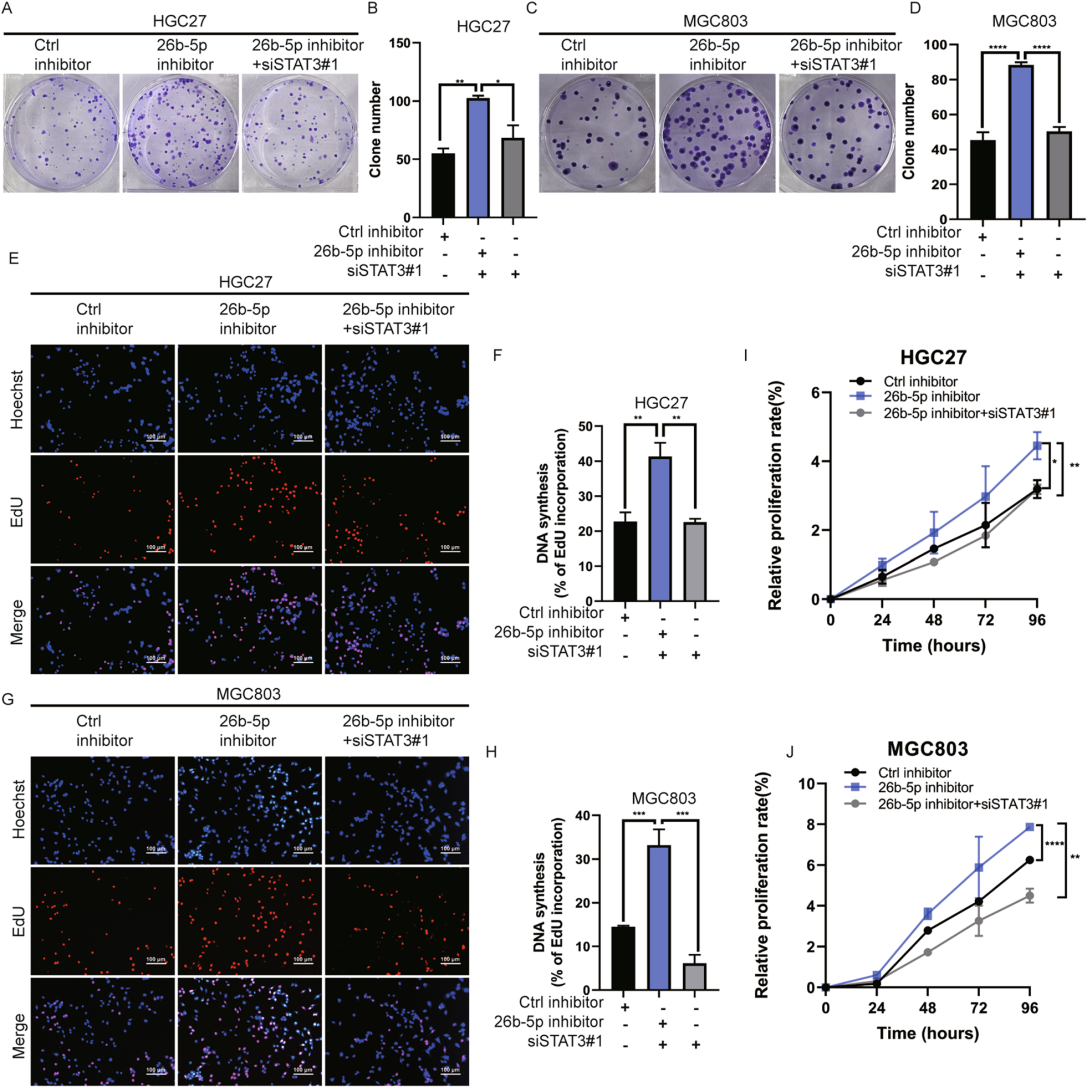
MiR-26b-5p modulates GC proliferation through STAT3. Colony formation **A**–**D**, EdU **E**–**H**, and CCK8 **I**–**J** assays were conducted in MGC803 or HGC27 cells transfected with miR-26b-5p inhibitor or miR-26b-5p inhibitor plus STAT3 siRNAs to examine the effect of STAT3 knockdown on the biologic function of miR-26b-5p. Quantitative data are shown as the mean ± SD of three independent experiments. *P < 0.05, **P < 0.01, ***P < 0.001, ****P < 0.0001

Therefore, we reasoned that miR-26b-5p might modulate the STAT3 signaling pathway by directly targeting PDE4B and CDK8. To elucidate the effect of miR-26b-5p on STAT3 signaling pathway activation, the total and phosphorylation levels of the key protein STAT3 were detected by Western blot. The result showed that miR-26b-5p upregulation suppressed activation of the STAT3 phosphorylation, while the inhibition of miR-26b-5p induced a significant increase in STAT3 phosphorylation (Fig. 6A–B). What’s more, IL-6-stimulated dose-dependent elevation of STAT3 phosphorylation was also negatively regulated by exogenous miR-26b-5p overexpression (Fig. 6F). Consistently, we further observed that miR-26b-5p mimics blocked the IL-6-induced nuclear translocation of STAT3 compared to IL-6-treated NC-transfectants control groups (Fig. 6C–D). Consistently with cell models, the in vivo results confirmed that miR-26b-5p negatively regulated STAT3 phosphorylation level*,* as detected by IHC staining (Fig. 6E). Moreover, the restoration of PDE4B or CDK8 expression reversed the decrease of STAT3 phosphorylation induced by overexpressing miR-26b-5p (Fig. 6G–H), while silencing PDE4B or CDK8 remarkably rescued the upregulation of p-STAT3 by miR-26b-5p knockdown (Fig. 6I–J). We next sought to determine whether STAT3 is a mediator accounting for cancer regulation of miR-26b-5p. We found that STAT3 silencing dramatically overturned the cancer-promoting effect of the miR-26b-5p inhibitor on cells (Fig. 7A–J). Taken together, these findings demonstrate that miR-26b-5p abrogates the STAT3 activation and STAT3-mediated cell growth by targeting PDE4B and CDK8.

### STAT3 transcriptionally suppresses miR-26b-5p

**Fig. 8:**
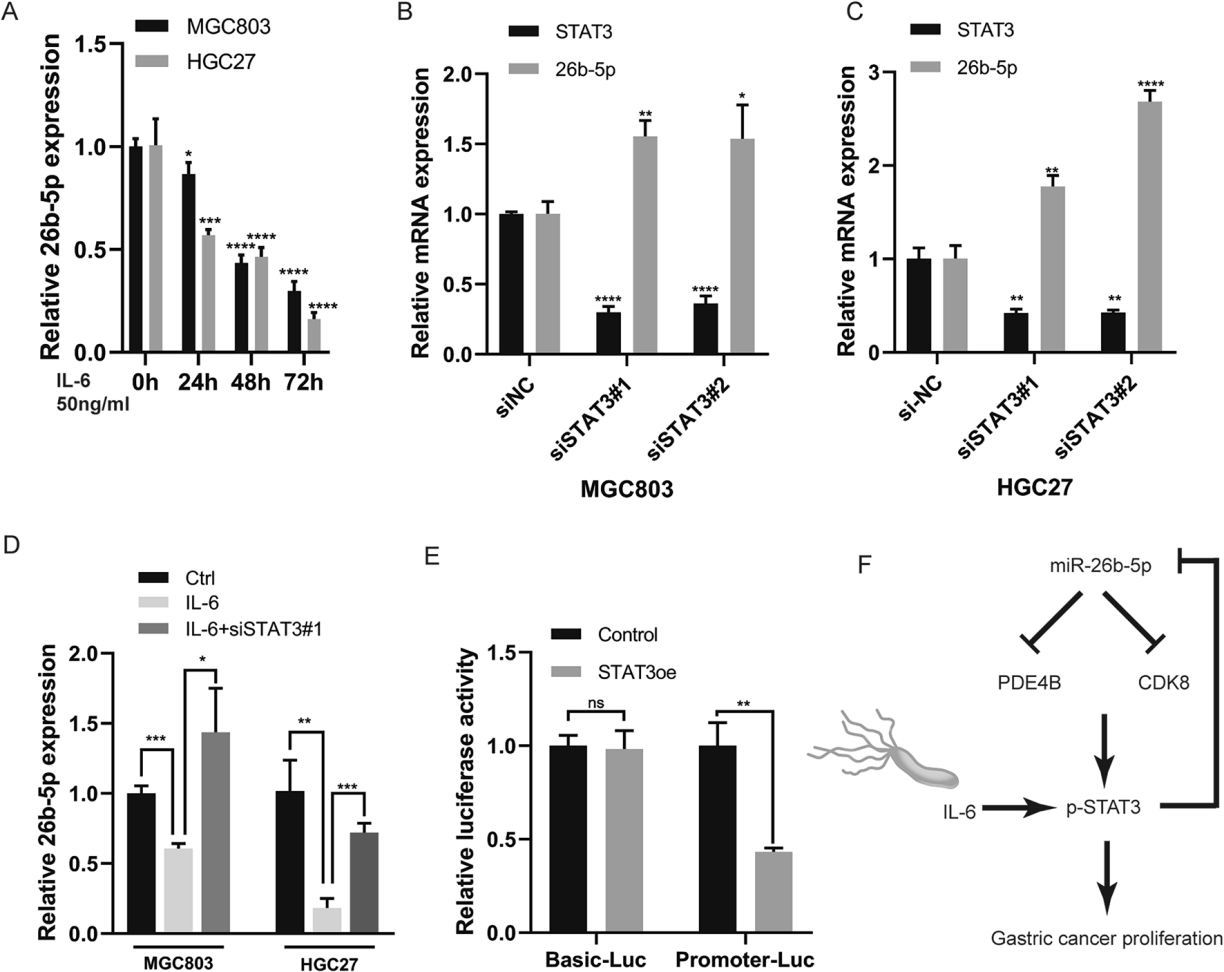
STAT3 transcriptionally suppresses miR-26b-5p. **A** MGC803 and HGC27 cells were treated with IL-6 at the concentration of 50 mg/ml for indicated times, and the expression of miR-26b-5p was detected by qRT-PCR. MGC803 **B** and HGC27 cells **C** were transfected with STAT3 siRNAs, and the expression of STAT3 and miR-26b-5p was examined using qRT-PCR. **D** MGC803 and HGC27 cells were transfected with STAT3 siRNAs or siNC and then treated with IL-6 (50 mg/ml). The expression of miR-26b-5p was detected by qRT-PCR. **E** HEK-293 T cells were co-transfected with pGL3-luciferase promoter plasmid containing the whole miR-26b-5p promoter region or pGL3-luciferase basic plasmid and STAT3 overexpression plasmids or control. The relative luciferase activities were determined 48 h after transfection. **F** The schematic model of miR-26b-5p-mediated GC proliferation. IL-6 induced by *H.pylor*i infection activates STAT3 which directly suppresses miR-26b-5p expression, resulting in the upregulation of miR-26b-5p’s targets, PDE4B and CDK8. The increase of PDE4B and CDK8 causes GC proliferation through STAT3 signaling. Quantitative data are shown as the mean ± SD of three independent experiments. *P < 0.05, **P < 0.01, ***P < 0.001, ****P < 0.0001

To further elucidate the connection between STAT3 and miR-26b-5p, we treated MGC803 and HGC27 with IL-6. It was found that the level of miR-26b-5p was decreased in a time-dependent manner after IL-6 treatment (Fig. 8A), while the silence of STAT3 induced the increase of miR-26b-5p expression (Fig. 8B–C). Additionally, STAT3 knockdown attenuated the inhibition of miR-26b-5p expression by IL-6 (Fig. 8D). Then we determined whether STAT3 directly regulated miR-26b-5p. miR-26b-5p is an intronic miRNA sharing promoter with its host gene CTDSP1 [30]. A series of potential binding sites of STAT3 was found in the miR-26b-5p promoter region predicted by Jaspar (Additional file 7: Table S1). We cloned the whole promoter region of miR-26b-5p and inserted it into the pGL3-luciferase reporter plasmids. The luciferase assay showed that exogenous STAT3 overexpression remarkably inhibited the luciferase activity of miR-26b-5p promoter plasmid, while no significant differences were observed in control groups. Together, these data suggest that STAT3 transcriptionally suppresses miR-26b-5p.

## Discussion

Evidence has established that the increase of inflammatory cytokines secretion, such as IL-6, and the arousal of chronic inflammation in the host body after *H. pylori* infection plays a crucial role in the transition of inflammation to GC [31–35]. The persistent chronic inflammation influences the cell cycle as well as apoptosis of gastric epithelial cells and eventually triggers gastric carcinogenesis. Therefore, it is necessary to find the possible biological targets in the long-term progression between *H. pylori*-induced inflammation and GC occurrence. Numerous studies have reported that miRNAs execute their regulatory functions in the development of inflammation-related cancers [36–38]. Here, we found that miR-26b-5p was downregulated under *H. pylori* infection as well as in GC tissues and cells, and low expression of miR-26b-5p was related to the poor outcome of patients. We then identified miR-26b-5p as a possible regulator which might connect chronic inflammation with GC.

It has been reported that miR-26b-5p, functioning as a tumor suppressor, regulates the carcinogenesis and oxaliplatin resistance of GC by targeting EZH2 [39, 40]. Also, the expression of miR-26b was lower in GC cells that are resistant to paclitaxel, suggesting that miR-26b might play a role in chemotherapy resistance [41]. In the present study, we observed that knockdown of miR-26b-5p promoted GC cell growth in vitro and in vivo, while restoration of miR-26b-5p was able to reverse such effects, which is consistent with previous studies. Our study identified that miR-26b-5p regulated GC progression by targeting PDE4B and CDK8 using bioinformatic analysis and a series of biochemical experiments. PDE4B exerts biological activities by controlling the degradation rate of cyclic adenosine monophosphate (cAMP). In recent years, PDE4B selective-targeted therapies have shown promising therapeutic perspectives in anti-inflammation and anti-cancer [42]. To our knowledge, there have been no reports about the biological effect of PDE4B in GC cell lines, and we proposed the oncogenic role of PDE4B in GC progression. In addition, numerous studies have supported that CDK8 is a carcinogen that regulates the malignant activities of tumors, including GC [43–45]. Notably, in our study, rescue experiments revealed that miR-26b-5p attenuated the proliferation of GC by directly interacting with PDE4B and CDK8, suggesting that the regulation of PDE4B and CDK8 by miR-26b-5p plays a key role in GC development.

As aforementioned, elevated IL-6 under *H. pylori* infection stimulates STAT3 activation via phosphorylation at tyrosine 705 (Y705) or serine 727 (S727) residues of STAT3. Constitutive activated STAT3 can then be transported into the nucleus to upregulate numerous STAT3 targets including Cyclin D1 and Bcl-2 [46, 47] which may result in out-of-control cell growth [48]. It reported that both Y705 and S727 phosphorylation of STAT3 was observed to be reduced by CDK8 inhibition [26–29]. Our study demonstrated that PDE4B and CDK8 linked miR-26b-5p and the STAT3 signaling pathway in GC. GSEA analysis revealed that PDE4B was positively related to the STAT3 signaling pathway. Western blot analysis showed that miR-26b-5p inhibition significantly activated the phosphorylation of STAT3, which, importantly, could be overturned by CDK8 or PDE4B overexpression. Also, in our functional studies, we found that STAT3 knockdown could effectively abrogate the effect of miR-26b-5p suppression and PDE4B or CDK8 overexpression on cell proliferation, indicating that the promotion of cell proliferation mediated by miR-26b-5p inhibition can be explained, at least partially, by activation of the downstream STAT3 signaling pathway. What’s more, restoration of miR-26b-5p impaired the STAT3 phosphorylation in response to IL-6 and blocked the translocation of STAT3 to the nucleus, indicating that miR-26b-5p may be a potential target for the treatment of IL-6/STAT3-mediated GC. Furthermore, a significant decrease of miR-26b-5p expression was observed in IL-6-treated GC cells, and STAT3 knockdown attenuated IL-6-stimulated miR-26b-5p reduction, indicating a negative regulation of IL-6/STAT3 and miR-26b-5p. Luciferase assay confirmed that STAT3 transcriptionally suppressed miR-26b-5p, thus forming the miR-26b-5p/STAT3 feedback loop, which provides a potential explanation of constitutive STAT3 activation in GC.

There are certainly some limitations in our study. We should detect the expression of miR-26b-5p in GC tissues with *H. pylori* infection to validate the clinical significance of miR-26b-5p in inflammation-related GC. Moreover, we did not conduct functional experiments to explore the effect of miR-26b-5p on GC cell proliferation with *H. pylori* infection due to limited experimental conditions. Furthermore, other downstream pathways regulated by miR-26b-5p, PDE4B, and CDK8 in GC progression remained to be investigated in the near future, such as Wnt/β-catenin [15, 44], NF-κB [14, 49], and Notch signaling pathways [50].

In conclusion, we found that miR-26b-5p was down-regulated by *H. pylori* infection and STAT3 transcription. MiR-26b-5p regulated the progression of GC by directly targeting PDE4B and CDK8 as well as mediating the downstream STAT3 signaling pathway, thus forming a miR-26b-5p/STAT3 feedback loop. We provide a basis for the future understanding of inflammatory signaling in GC development and suggested that miR-26b-5p, PDE4B, and CDK8 may be promising biomarkers for clinical therapies for inflammation-mediated GC.

## Supplementary Information

**Additional file 1: Figure S1.** (A-B) ROC-curves and AUC-scores of miR-26b-5p in TCGA-STAD dataset and GSE30070.

**Additional file 2: Figure S2.** (A) Transfection efficiency of miR-26b-5p mimics in MGC803 and HGC27 cells using qRT-PCR. CCK8 (B-C), colony formation (D-E), and EdU (F–H) assays were used to determine the effect of miR-26b-5p mimics transfection on the proliferation of MGC803 and HGC27 cells. (I) Cell cycle analysis was determined in MGC803 and HGC27 cells transfected with miR-26b-5p mimics. Quantitative data are shown as the mean ± SD of three independent experiments. *P < 0.05, **P < 0.01, ***P < 0.001, ****P < 0.0001 (Student’s t-test).

**Additional file 3: Figure S3.** (A) Transfection efficiency of miR-26b-5p inhibitor in BGC823 using qRT-PCR. (B-D) CCK8 and colony formation was utilized to explore the effect of miR-26b-5p silencing on the proliferation of BGC823 cells.

**Additional file 4: Figure S4.** (A) Schematic picture of the three-step approach used to determine targets of miR-26b-5p. (B) Venn diagram of the potential targets of miR-26b-5p. (C) Correlation analysis of the expression of genes determined in (A) and the expression of miR-26b-5p. (D) The expression of genes determined in (A) in GC tissue compared to normal gastric tissue based on the TCGA database.

**Additional file 5: Figure S5.** (A-D) EdU, colony formation, and CCK8 analysis of MGC803 cells transfected with siPDE4B or siCDK8. (E–H) colony formation, EdU and CCK8 assays were used to assess the proliferation capacity of MGC803 cells transfected with Ctrl inhibitor + si-NC, 26b-5p inhibitor + si-NC, 26b-5p inhibitor + siCDK8, or 26b-5p inhibitor + siPDE4B. Quantitative data are shown as the mean ± SD of three independent experiments. *P < 0.05, **P < 0.01, ***P < 0.001, ****P < 0.0001 (Student’s t-test).

**Additional file 6: Figure S6.** CCK8 (A), colony formation (C-D), and EdU (G, J) analysis of MGC803 cells transfected with PDE4B overexpression plasmids or PDE4B overexpression plasmids plus siSTAT3. CCK8 (B), colony formation (E–F), and EdU (H-I) analysis of MGC803 cells transfected with CDK8 overexpression plasmids or CDK8 overexpression plasmids plus siSTAT3. Quantitative data are shown as the mean ± SD of three independent experiments. *P < 0.05, **P < 0.01, ***P < 0.001, ****P < 0.0001 (Student’s t-test).

**Additional file 7: Table S1.** Predicted STAT3 binding sites in miR-26b-5p promoter region. **Table S2.** The sequences of siRNA, miRNA mimics and miRNA inhibitor.

## Data Availability

The datasets supporting the conclusions of this article are included within the article.

## Abbreviations

GC: Gastric cancer
TCGA: The Cancer Genome Atlas
GEO: Gene expression omnibus
*H. pylori*: *Helicobacter Pylori*
qRT-PCR: Quantitative real-time PCR
CDK8: Cyclin dependent kinase 8
PDE4B: Phosphodiesterase 4B
STAT3: Signal transducer and activator of transcription 3
IL: Interleukin
JAK: Janus Kinase
miRNAs: MicroRNAs
3’UTR: 3’-Untranslated regions
NF-κB: Nuclear factor kappa-B
TAK1: TGF-beta activated kinase 1
TAB3: TGF-beta activated kinase 1 binding protein 3
CCK8: Cell Counting Kit-8
EdU: 5-Ethynyl-2’-deoxyuridine
IHC: Immunohistochemistry
cAMP: Cyclic adenosine monophosphate
MOI: Multiplicity of infection
GSEA: Gene set enrichment analysis
IF: Immunofluorescence
MOI: Multiplicity of infection
SD: Standard deviation
FC: Foldchange
AUC: Area under the curve
ANOVA: One-way analysis of variance
STAD: Stomach adenocarcinoma
RT: Room temperature
FL: Firefly luciferase
RL: Renilla luciferase
siRNA: Small interfering RNA
PBS: Phosphate buffered saline
GES1: Normal gastric epithelium cells
HEK-293 T: Human embryonic kidney cells
JAK2: Janus kinase 2
DLGAP1-AS1: LncRNA DLGAP1 antisense RNA 1
GAPDH: Glyceraldehyde-3-phosphate dehydrogenase
