# Supplementary figures and images for: Loss of miR-26b-5p promotes gastric cancer progression via miR-26b-5p-PDE4B/CDK8-STAT3 feedback loop
Source: J Transl Med. 2023 Feb 3;21:77. doi: 10.1186/s12967-023-03933-x (PMC9898947; doi:10.1186/s12967-023-03933-x)

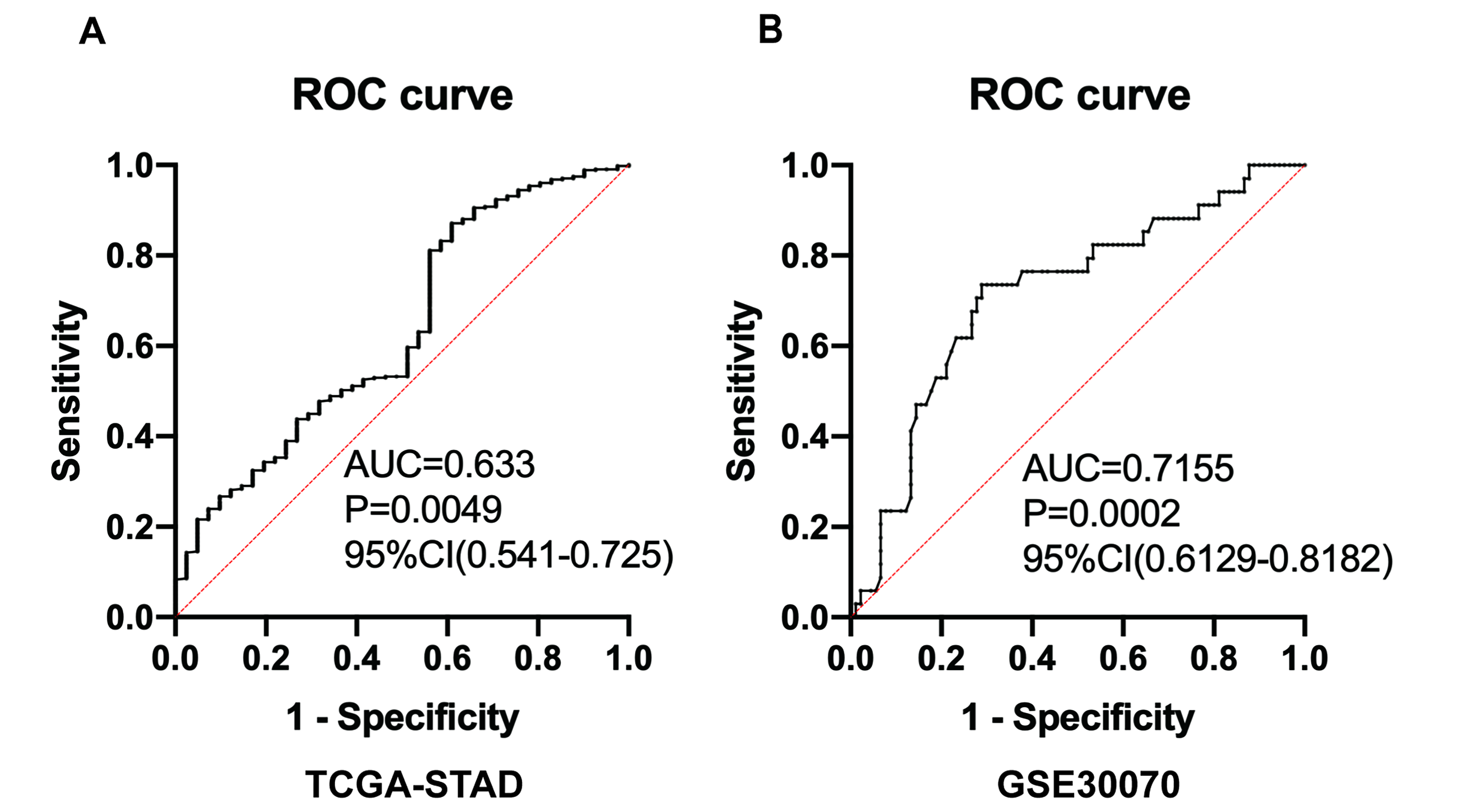

Supplement: Supplementary file 1 — Additional file 1: Figure S1. (A-B) ROC-curves and AUC-scores of miR-26b-5p in TCGA-STAD dataset and GSE30070. [file 12967_2023_3933_MOESM1_ESM.tif]

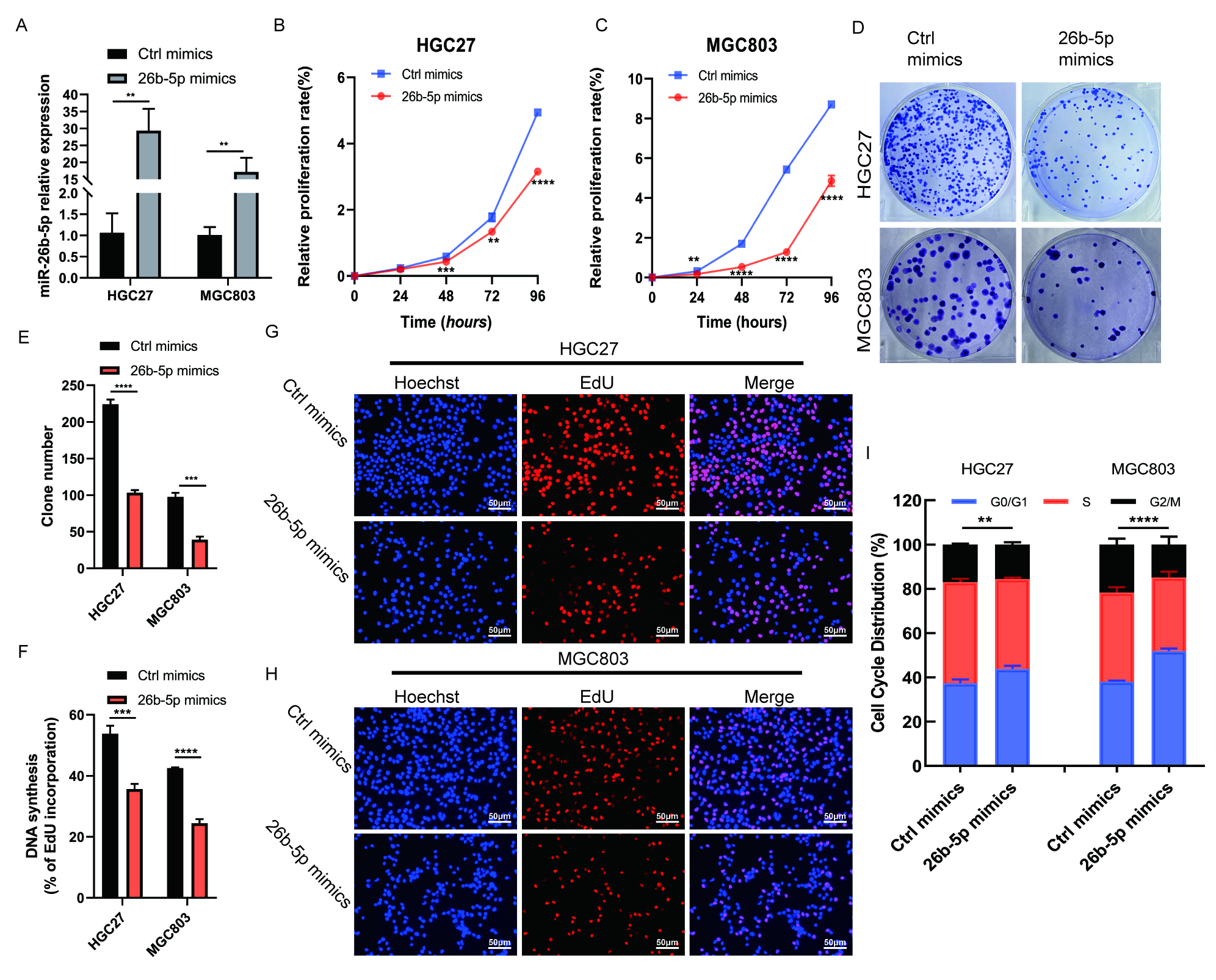

Supplement: Supplementary file 2 — Additional file 2: Figure S2. (A) Transfection efficiency of miR-26b-5p mimics in MGC803 and HGC27 cells using qRT-PCR. CCK8 (B-C), colony formation (D-E), and EdU (F–H) assays were used to determine the effect of miR-26b-5p mimics transfection on the proliferation of MGC803 and HGC27 cells. (I) Cell cycle analysis was determined in MGC803 and HGC27 cells transfected with miR-26b-5p mimics. Quantitative data are shown as the mean ± SD of three independent experiments. *P < 0.05, **P < 0.01, ***P < 0.001, ****P < 0.0001 (Student’s t-test). [file 12967_2023_3933_MOESM2_ESM.tif]

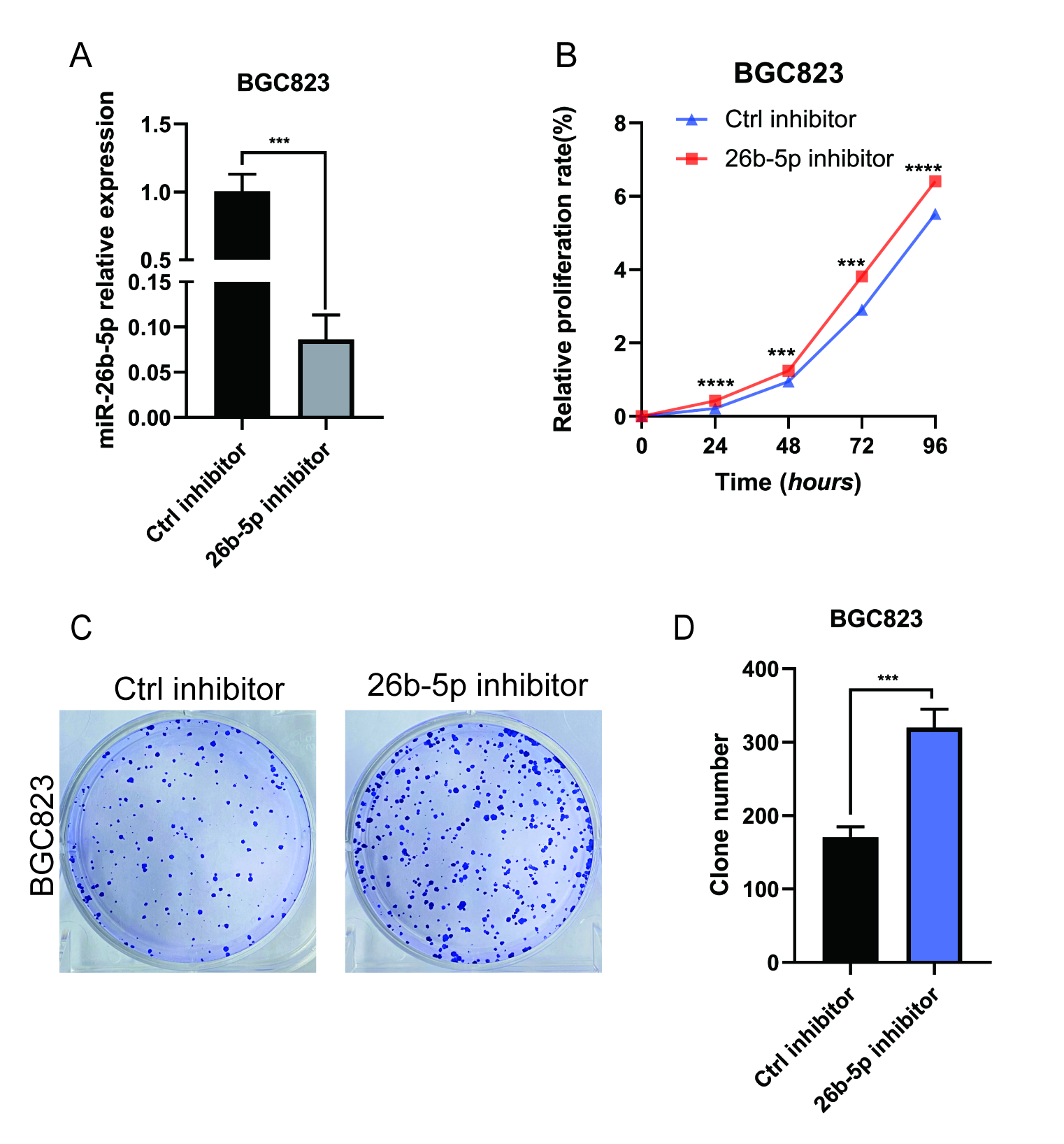

Supplement: Supplementary file 3 — Additional file 3: Figure S3. (A) Transfection efficiency of miR-26b-5p inhibitor in BGC823 using qRT-PCR. (B-D) CCK8 and colony formation was utilized to explore the effect of miR-26b-5p silencing on the proliferation of BGC823 cells. [file 12967_2023_3933_MOESM3_ESM.tif]

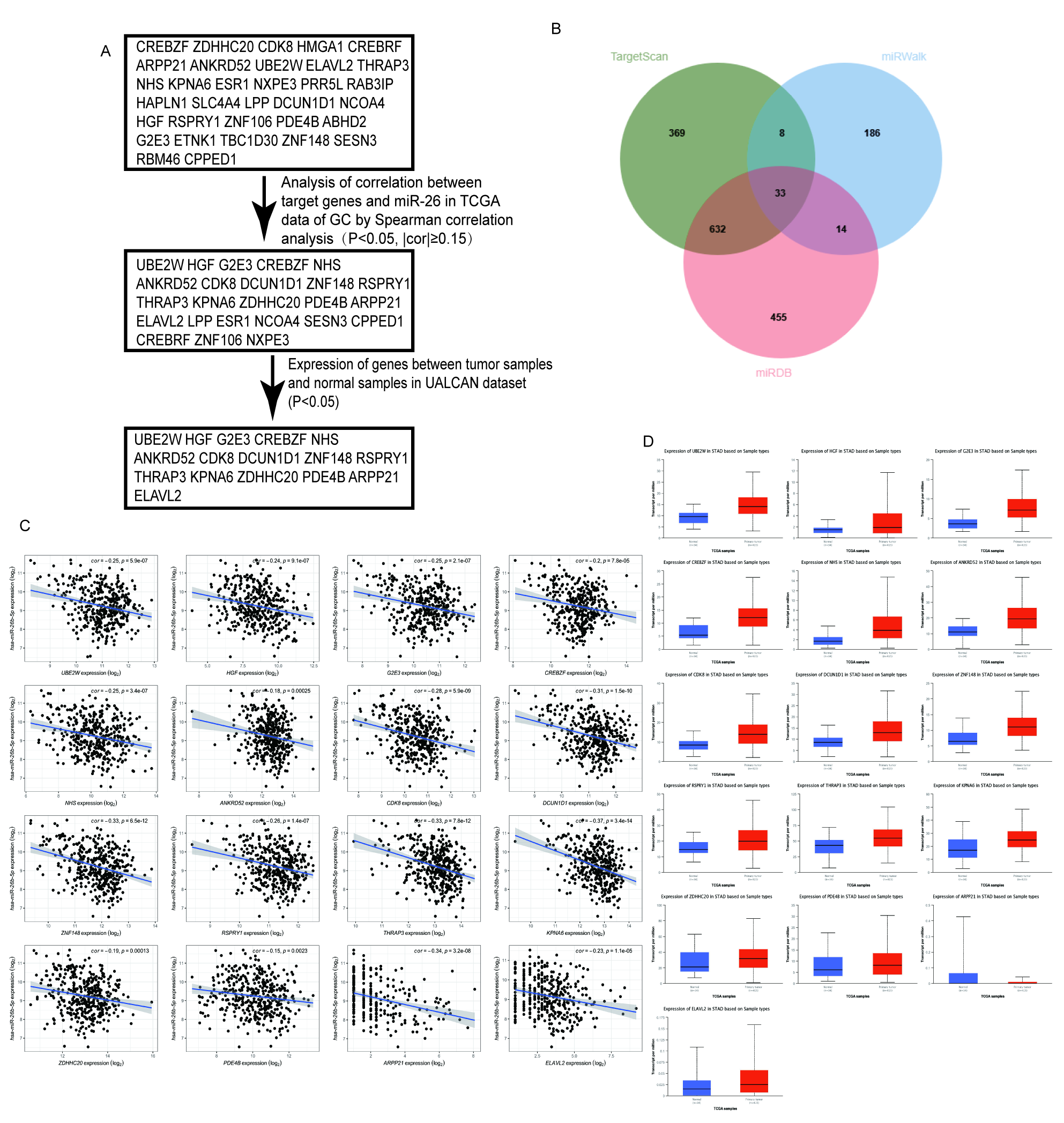

Supplement: Supplementary file 4 — Additional file 4: Figure S4. (A) Schematic picture of the three-step approach used to determine targets of miR-26b-5p. (B) Venn diagram of the potential targets of miR-26b-5p. (C) Correlation analysis of the expression of genes determined in (A) and the expression of miR-26b-5p. (D) The expression of genes determined in (A) in GC tissue compared to normal gastric tissue based on the TCGA database. [file 12967_2023_3933_MOESM4_ESM.tif]

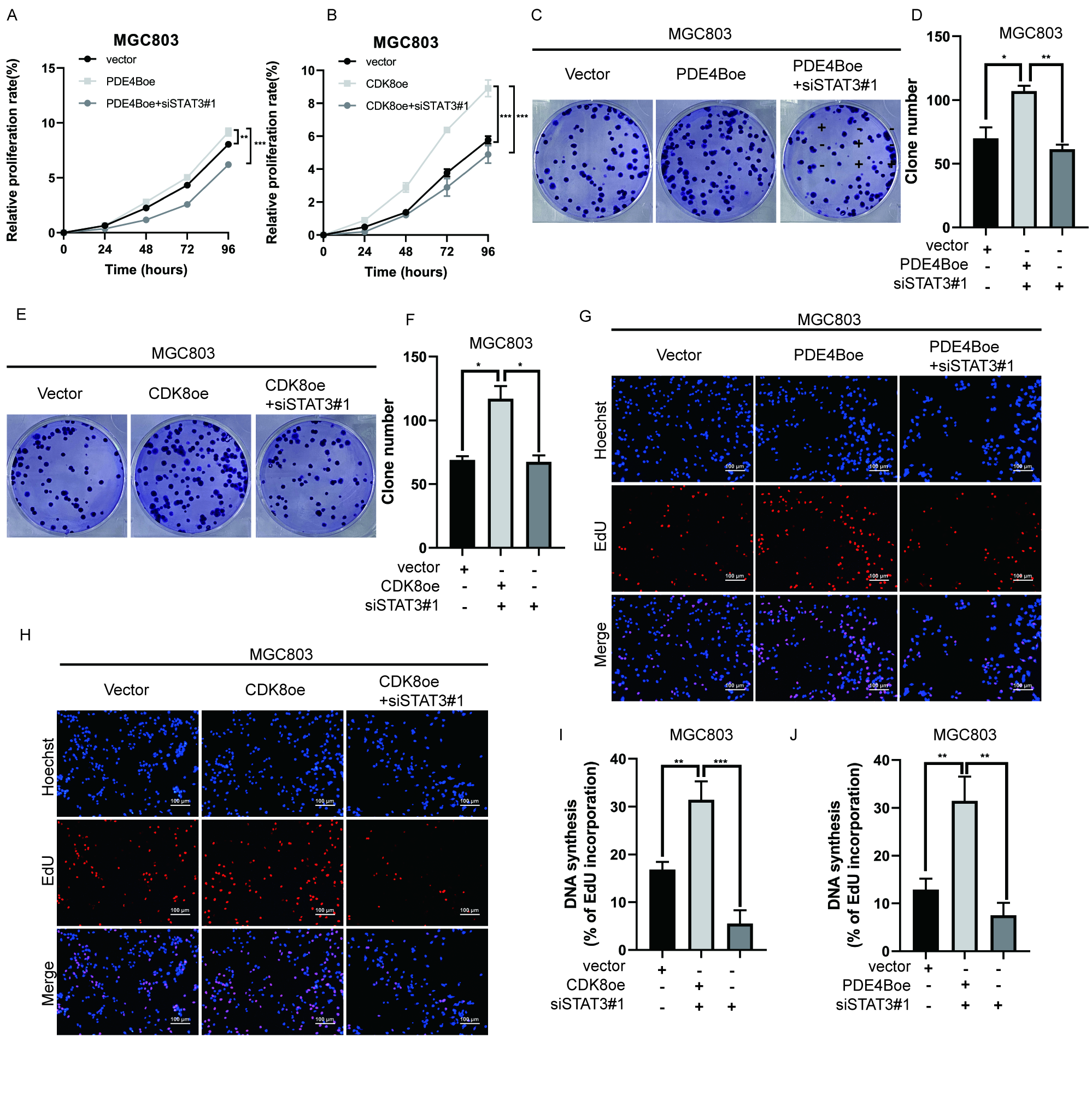

Supplement: Supplementary file 5 — Additional file 5: Figure S5. (A-D) EdU, colony formation, and CCK8 analysis of MGC803 cells transfected with siPDE4B or siCDK8. (E–H) colony formation, EdU and CCK8 assays were used to assess the proliferation capacity of MGC803 cells transfected with Ctrl inhibitor + si-NC, 26b-5p inhibitor + si-NC, 26b-5p inhibitor + siCDK8, or 26b-5p inhibitor + siPDE4B. Quantitative data are shown as the mean ± SD of three independent experiments. *P < 0.05, **P < 0.01, ***P < 0.001, ****P < 0.0001 (Student’s t-test). [file 12967_2023_3933_MOESM5_ESM.tif]

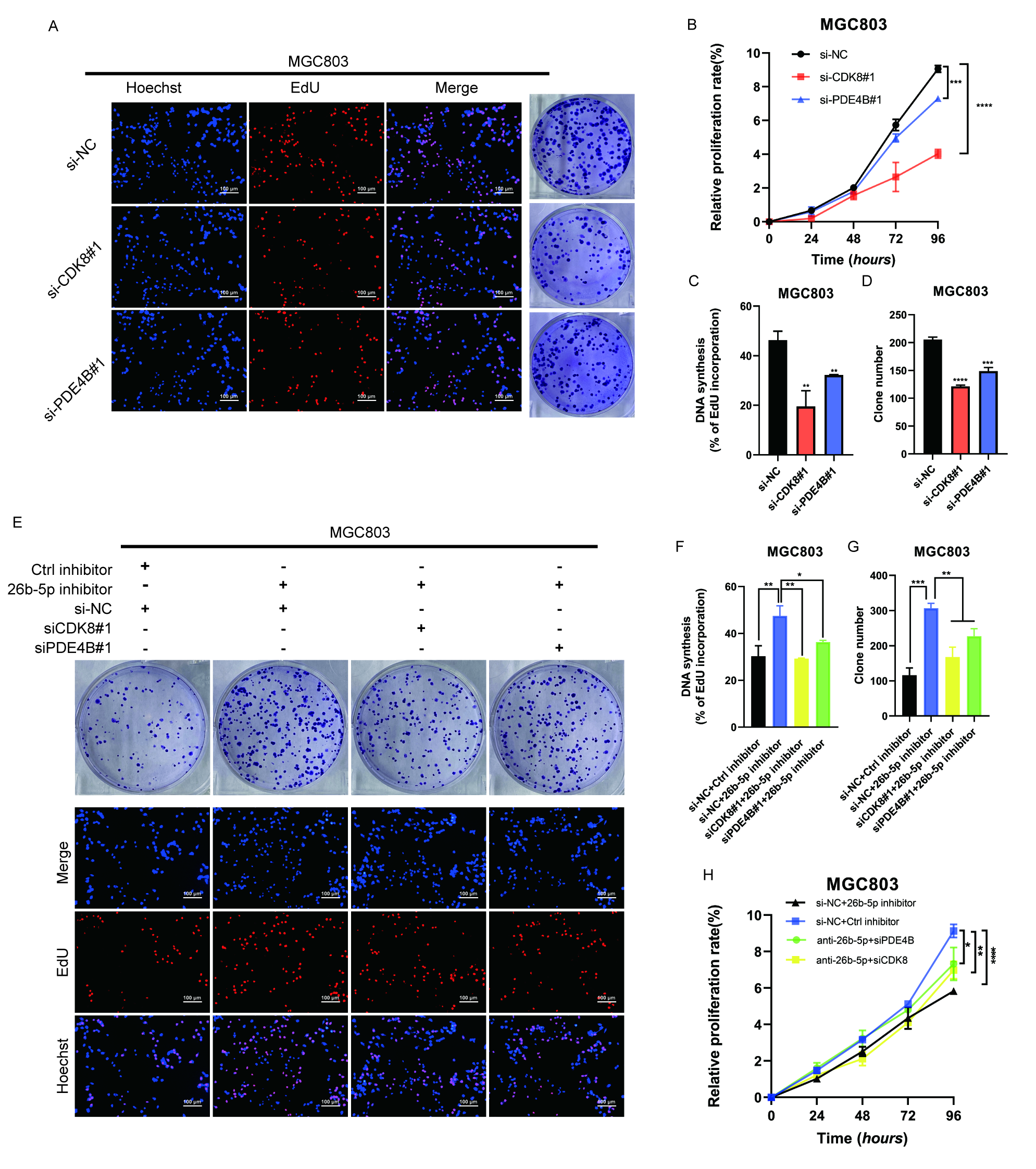

Supplement: Supplementary file 6 — Additional file 6: Figure S6. CCK8 (A), colony formation (C-D), and EdU (G, J) analysis of MGC803 cells transfected with PDE4B overexpression plasmids or PDE4B overexpression plasmids plus siSTAT3. CCK8 (B), colony formation (E–F), and EdU (H-I) analysis of MGC803 cells transfected with CDK8 overexpression plasmids or CDK8 overexpression plasmids plus siSTAT3. Quantitative data are shown as the mean ± SD of three independent experiments. *P < 0.05, **P < 0.01, ***P < 0.001, ****P < 0.0001 (Student’s t-test). [file 12967_2023_3933_MOESM6_ESM.tif]
