# Supplementary material for: Loss of miR-26b-5p promotes gastric cancer progression via miR-26b-5p-PDE4B/CDK8-STAT3 feedback loop
Source: J Transl Med. 2023 Feb 3;21:77. doi: 10.1186/s12967-023-03933-x (PMC9898947; doi:10.1186/s12967-023-03933-x)
Supplement: Supplementary file 7 — Additional file 7: Table S1. Predicted STAT3 binding sites in miR-26b-5p promoter region. Table S2. The sequences of siRNA, miRNA mimics and miRNA inhibitor. [file 12967_2023_3933_MOESM7_ESM.docx]

| Score | Relative score | Start | End | Strand | Predicted site sequence |
| --- | --- | --- | --- | --- | --- |
| 9.570445 | 0.913496406 | 2041 | 2051 | + | TTTCTTGAAAC |
| 8.657998 | 0.902442518 | 316 | 326 | + | TTTCTTAAAAG |
| 8.1099415 | 0.895803056 | 296 | 306 | + | TTTATGAGAAG |
| 7.6001954 | 0.88962771 | 2040 | 2050 | - | TTTCAAGAAAG |
| 7.244311 | 0.885316327 | 316 | 326 | - | CTTTTAAGAAA |
| 6.5962195 | 0.87746499 | 1765 | 1775 | + | CGTCTGGGAAT |
| 5.499449 | 0.864178101 | 531 | 541 | + | CCTCCTGGAAG |
| 5.451496 | 0.863597176 | 1110 | 1120 | - | CTTCCTGGAGG |
| 5.3409944 | 0.862258496 | 2041 | 2051 | - | GTTTCAAGAAA |

**Additional Table S1.** Predicted STAT3 binding sites in miR-26b-5p promoter region.

**Additional Table S2.**The sequences of siRNA, miRNA mimics and miRNA inhibitor.

| Name | Sequence |
| --- | --- |
| miR-26b-5p mimics | sense（5’-3’）：UUCAAGUAAUUCAGGAUAGGU |
|  | antisense（5’-3’）：CUAUCCUGAAUUACUUGAAUU |
| miR-26b-5p inhibitor | ACCUAUCCUGAAUUACUUGAA |
| si-CDK8#1 | GGAGCAAGGCATTATACCA |
| siCDK8#2 | GTCGGCATGTAGAGAAATA |
| si-PDE4B#1 | CCTACATGATGACTTTAGA |
| siPDE4B#2 | GCGTTCTTCTCCTAGACAA |
| si-STAT3#1 | GGCGTCCAGTTCACTACTA |
| si-STAT3#2 | AGACCCGTCAACAAATTAA |
